# Effectiveness of Telemedicine Nursing Interventions in the Management of Overweight and Obesity in Adults: A Systematic Review and Meta-Analysis

**DOI:** 10.1007/s13679-025-00655-7

**Published:** 2025-07-30

**Authors:** Emanuele Colaone, Elisa Belluzzi, Assunta Pozzuoli, Enrico Roma, Elena Bortolato, Carlo Biz, Pietro Ruggieri

**Affiliations:** 1 Department of Medicine, University of Padova, Via Giustiniani 3, Padova, 35128 Italy; 2 Musculoskeletal Pathology and Oncology Laboratory, Department of Surgery, Oncology and Gastroenterology (DiSCOG), University of Padova, Via Giustiniani 3, Padova, 35128 Italy; 3 Orthopedics and Orthopedic Oncology, Department of Surgery, Oncology and Gastroenterology (DiSCOG), University-Hospital of Padova, Via Giustiniani 3, Padova, 35128 Italy; 4 Centre for Mechanics of Biological Materials, University of Padova, Padova, 35131 Italy; 5 Department of Statistical Science “Paolo Fortunati”, University of Bologna, Bologna, Italy; 6 Department of Business and Economics, Universitat Pompeu Fabra, Barcelona, 08005 Spain; 7 Data Science Center, Barcelona School of Economics, Barcelona, 08005 Spain

**Keywords:** Nursing, Telemedicine, Obesity, Overweight, Systematic review, Meta-analysis, Remote healthcare

## Abstract

**Background:**

Obesity is a growing global health issue, linked to chronic conditions like type 2 diabetes, hypertension, and cardiovascular diseases. Nursing staff could play a significant role in managing obesity, with telemedicine being a potentially effective tool for monitoring weight and caloric intake. However, the effectiveness of telemedicine-based nursing interventions in managing obesity and overweight remains unclear.

**Objective:**

This systematic review with meta-analysis aimed to evaluate the effectiveness of nursing telemedicine interventions in managing overweight and obesity in adults.

**Method:**

This review followed PRISMA guidelines and involved a literature search in Scopus, PubMed, and Web of Science. Eligible studies were in English, involved adults with a BMI over 25, and evaluated nursing-led telemedicine interventions (non-randomized and randomized controlled studies). Study quality was assessed using the Down and Black checklist. A random-effects model was used for the meta-analysis.

**Results:**

Four moderate-quality articles were included. The nursing-led telemedicine interventions, delivered through digital platforms, mobile apps, and remote monitoring, focused on health education and motivational strategies to promote self-regulation and dietary improvements. The meta-analysis showed that, compared to traditional approaches, nursing-led interventions resulted in an average weight loss of 2.59 kg (95% CI: − 3.09 to − 2.08), a reduction of 1.05 kg/m² in BMI (95% CI: − 1.50 to − 0.60), and a decrease of 2.52 cm in waist circumference (95% CI: − 2.96 to − 2.09).

**Conclusion:**

Nursing telemedicine interventions are effective in promoting short-term weight loss and lifestyle changes. However, further research is needed to assess long-term effects and the impact of different interventions.

**Supplementary Information:**

The online version contains supplementary material available at 10.1007/s13679-025-00655-7.

## Introduction

Obesity is currently recognized as a disease by multiple organizations, including the World Health Organization (WHO) [1]. This condition is characterized by an excessive accumulation of body fat, which can negatively impact health. It is a highly complex chronic disease and a significant risk factor for various other illnesses, primarily cardiovascular diseases, type 2 diabetes, hypertension, osteoarthritis, and certain types of cancer [2, 3].

Obesity is commonly defined using the Body Mass Index (BMI), calculated as weight in kilograms divided by height in meters squared (kg/m²). The WHO classifies individuals as overweight with a BMI between 25 and 29.9, and as obese if their BMI is 30 or higher. Obesity is further categorized into three classes: Class 1 obesity (BMI 30–34.9), Class 2 obesity (BMI 35–39.9), and Class 3 obesity (BMI ≥ 40) [4].

Obesity is a multifactorial condition, influenced by genetic predispositions and environmental factors, such as high-calorie diets and sedentary lifestyles [5, 6]. Most cases of obesity are polygenic, involving multiple genetic mutations that increase fat accumulation, while rare cases of monogenic obesity lead to severe obesity beginning in early childhood [7]. Socioeconomic factors also play a critical role, with higher obesity rates observed among individuals with lower educational attainment and income levels [8].

Obesity rates have nearly tripled globally since 1975, with 13% of adults now obese, more commonly among women (15%) than men (11%). Over 650 million people are obese, and over 1.9 billion adults (39% of the global population) are overweight. Projections indicate that by 2030, 86% of U.S. adults will be overweight or obese, and 2.3 billion people globally classified as overweight [4, 9]. This escalating global health challenge has profound implications for both individual well-being and healthcare systems, with an estimated economic burden of $2 trillion—equivalent to 2.8% of the world’s gross domestic product [4]. Addressing obesity has become a priority for health professionals and policymakers, who urgently need effective strategies to prevent and manage it, and reduce the burden on healthcare systems [10].

Obesity is also recognized as a disease within the nursing profession. The North American Nursing Diagnosis Association International (NANDA-I), includes related diagnoses such as “risk of overweight”, “overweight”, and “obesity”, each with specific codes [11]. These diagnoses are integrated into standardized nursing taxonomies, linked to measurable health outcomes in the Nursing Outcomes Classification (NOC) [10] and to corresponding actions in the Nursing Interventions Classification (NIC) [9].

Current obesity treatments in healthcare settings encompass dietary, behavioural, pharmacological, and surgical approaches. Effective management requires a multidisciplinary, personalized strategy tailored to individual needs, with the primary goals of reducing body weight and improving overall health outcomes [12]. Among emerging interventions, digital tools—such as smartphone applications and fitness trackers—offer support by monitoring caloric intake and physical activity, helping to sustain motivation and track progress. A recent systematic review demonstrated that eHealth approaches are effective in weight loss in adults with overweight or obesity [13]. Telemedicine has gained traction for delivering behavioural support and nutritional counselling, enhancing the accessibility, personalization, and cost-effectiveness of care while addressing geographical and logistical barriers [5, 7]. Moreover, telemedicine interventions have been demonstrated to be promising in obesity management [8], as documented by several systematic reviews [12, 14–16].

Nurse-led interventions have been found to be promising in the prevention and treatment of paediatric overweight and obesity [17]. Nurses could play a crucial role in obesity management by addressing its psychological, nutritional, behavioural, and pharmacological dimensions. They can offer ongoing guidance, support, and motivation to patients, while actively collaborating with other healthcare professionals to ensure comprehensive care. In addition, nursing interventions can be sustained over time, promoting long-term weight management and improved health outcomes [18, 19].

Despite the growing evidence supporting the effectiveness of nursing telemedicine in various chronic conditions, including diabetes management, hypertension control, and post-surgical follow-up [20–22], its application to adult overweight and obesity remains largely unexplored, and no systematic reviews with meta-analysis have been published.

Therefore, the aim of this study was to evaluate the effectiveness of telemedicine-based nursing interventions in managing overweight and obesity in adult patients through a systematic review with meta-analysis of the literature. Specifically, it examined the effectiveness of telemedicine in assisting nurses with patient weight management, promoting lifestyle changes, and enabling long-term patient monitoring.

## Materials and Methods

### Search Strategy

This systematic review and meta-analysis was conducted following the guidelines outlined in the “Preferred Reporting Items for Systematic Reviews and Meta-Analyses” (PRISMA-2020) framework [23]. The review was registered on the Open Science Framework (OSF) with DOI: 10.17605/OSF.IO/GQ6WR.

A comprehensive literature search was conducted in August 2024 across three major databases - Scopus, Web of Science and Pubmed - using title-based filters. The keywords used for this review included *nursing*,* obesity*,* overweight*,* body weight*,* body mass index*,* BMI*,* weight*,* and nurse-led intervention*, combined with the Boolean operators “AND” and “OR” The final search string was as follows: ((overweight OR obes* OR “body weight” OR (body AND mass AND index) OR weight OR BMI) AND ((nurse-led AND intervention*) OR nurs*)).

### Inclusion and Exclusion Criteria

The review included only original research articles written in English, without temporal restrictions. The PICO framework was applied as follows: P (Population): Adult patients (≥ 18 years) who were overweight or obese (BMI ≥ 25); I (Intervention): Telemedicine nursing interventions; C (Comparison): patients receiving general care/traditional nursing interventions; O (Outcome): the primary outcome was weight change, while secondary outcomes included BMI and waist circumference (WC)/abdominal circumference.

Eligible studies were randomised and non-randomized clinical studies that reported weight measurements, changes in BMI or WC before and after the intervention. Studies had to compare telemedicine nursing interventions with either traditional nursing interventions or basic care. Qualitative studies and non-original research (e.g. literature reviews, editorials, comments, case reports, books, opinions, conference abstracts, or retracted articles) were excluded. Additionally, studies were excluded if they involved participants under 18 years old, had a BMI under 25, lacked nursing interventions, or did not report changes in BMI or weight after the intervention.

### Study Selection

The results obtained from the three databases were downloaded and imported into Zotero, a reference management software. Duplicate records were identified and removed. Following this, the remaining studies underwent a two-step screening process. Initially, titles and abstracts were reviewed to identify potentially eligible studies. Full-text screening was then conducted to determine final eligibility based on the inclusion and exclusion criteria.

Two independent reviewers conducted the search for relevant studies using the outlined strategy. In cases where eligibility was unclear, a third reviewer was consulted to reach a consensus.

### Data Extraction

Data from the included studies were extracted using a predefined data extraction form, capturing key details such as study design, authors, population characteristics, type of intervention, study description, and outcomes (before and after the intervention).

### Quality Assessment

To assess the methodological quality of the selected studies, the Downs and Black checklist [24] was used. This tool assigns a maximum score of 32 points, with higher values indicating better quality. The classification is as follows: high quality, 24–32 points; moderate quality, 15–23 points; low quality, ≤ 14 points.

### Statistical Analysis

All the included studies employed a pretest-posttest control group design. The effect size was defined as.

$\:\mu\:=\:\left({\mu\:}_{E,\:post\:}-{\mu\:}_{E,\:pre}\right)-\:\left({\mu\:}_{C,\:post\:}-{\mu\:}_{C,\:pre}\right),\:$ where $\:{\mu\:}_{E,\:pre}$ and $\:{\mu\:}_{E,\:post}$ represent the pretest and posttest means for the experimental group, and $\:{\mu\:}_{C,\:pre}$and $\:{\mu\:}_{C,\:post}$post are the corresponding means for the control group. This effect size is interpretable in the unit of measurement of the outcome of interest. A separate random-effects model was estimated for each outcome, as information on the correlations between outcomes was not available. Due to the limited number of studies, a second-order likelihood method based on Skovgaard’s statistic [25] was applied to improve the accuracy of the results. The findings were compared with those obtained using the classical method. Prediction intervals were not reported, as the square root of the heterogeneity parameter $\:{\tau\:}^{2\:}$ can be interpreted on the scale of the outcome. The statistical analyses were conducted in the R (R core Team) [26] statistical environment using the metafor [27] and metalik [25] packages. The significance level was set to $\:\alpha\:=0.05\:$.

## Results

### Literature Search

**Fig. 1:**
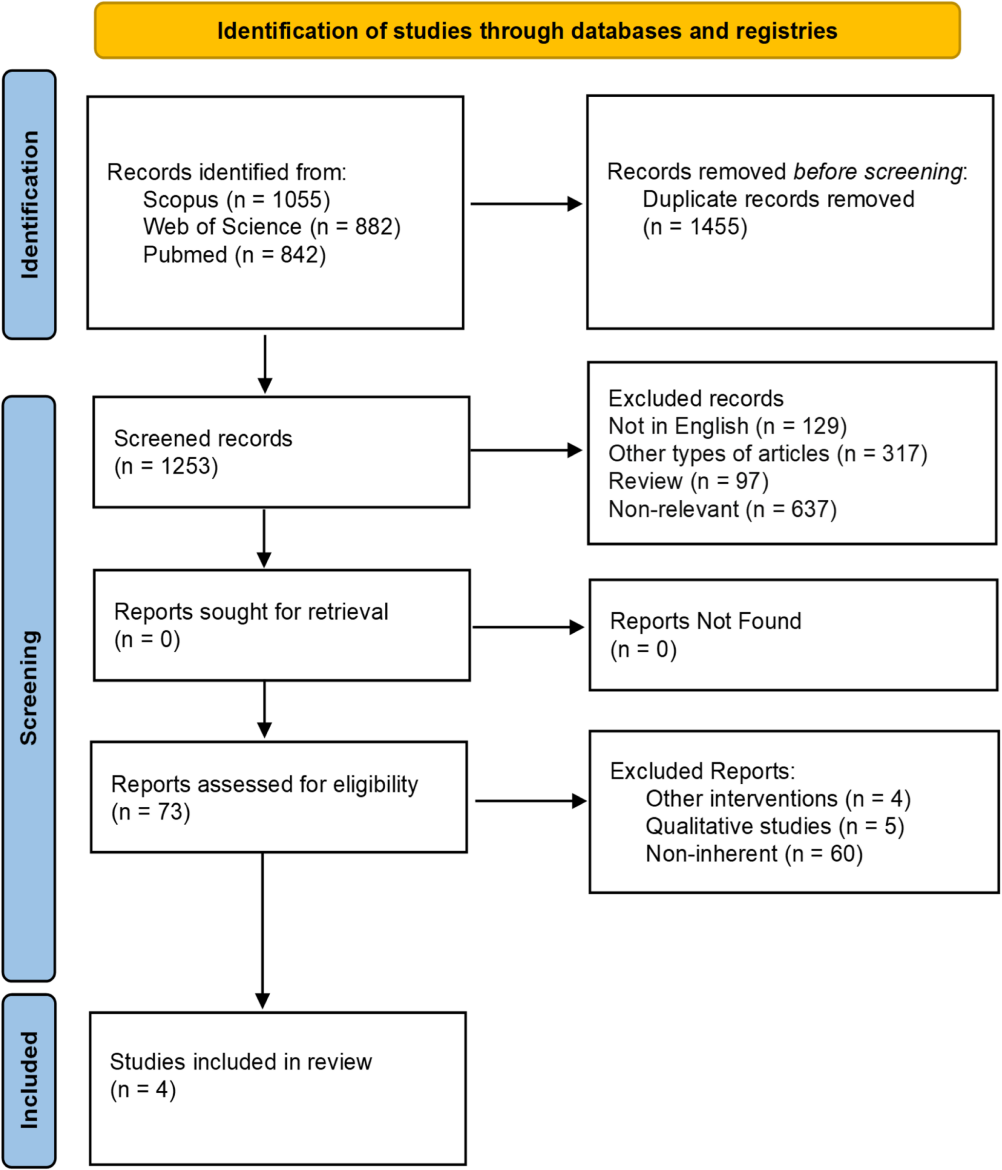
Flowchart of the search strategy according to PRISMA 2020 guidelines

A total of 2,779 records were initially identified (Fig. 1). After removing duplicates, 1,253 records were screened. Based on inclusion and exclusion criteria, 73 full text-articles were assessed for eligibility. Of these, 69 were excluded, resulting in four articles being included in the review [28–31].

### Studies Features

The characteristics of the four included studies are summarized in Table 1, which presents three randomized controlled trials (RCTs) and one quasi-experimental study.

**Table 1:** Characteristics of the studies included

Author (Publication year)	Study design	Country	Group description	Type of intervention	Study description	Number of patients	Age, years Mean ± SD	Duration of intervention
García-Rodríguez et al. (2024)	Quasi-experimental study	Tenerife (Spain)	Overweight or obese adults; age 18–45 years	Phone calls or emails, web support	CG: traditional method of monitoring by nurses. IG: telehealth-based platform (healthy lifestyle advice)	Total = 66 CG = 33 IG = 33	CG = 39.0 ± 5.6 IG = 34.1 ± 8.8	3 months
Krishnasamy et al. (2024)	RCT	Puducherry (India)	Obese adults; BMI > 25 age 18–50 years	Phone calls, video calls, texts, home visits, and use of fit-bit bands	CG: only basic care IG: telematic and face-to-face counseling and encouragement from nurses Both groups: fit-bit bands to monitor the various parameters.	Total = 88 CG = 44 IG = 44	Total = 38.04 ± 7.53	16 weeks
Little et al. (2016)	RCT	England	Adults ≥ 18 years; BMI ≥ 30	website, face-to-face and telematic nursing consultations	CG: website with information POWeR + F: website for selecting a meal plan and setting a calorie deficit; face-to-face meetings with nurse (3 times in the first 3 months and 4 in the following 3 months) POWeR + R: website; possibility to contact their nurse by phone or email (3 times in the first 3 months and 2 times in the following 3 months)	Total = 818 CG = 279 POWeR + F = 269 POWeR + *R* = 270	CG = 52.69 ± 13.25 POWeR + F = 53.70 ± 13.21 POWeR + *R* = 54.74 ± 12.95	12 months
Palmeira et al. 2019	RCT	Salvador, Bahia, Brazil	Women aged 18–60 years; BMI ≥ 25; at least one visit to this clinic in the past 12 months	Remote monitoring via phone calls	CG: routine FU consultations group IG: routine consultations, remote monitoring via phone calls.	Total = 101 females CG = 50 IG = 51	Total = 47.8 ± 9.0 CG = 47.5 ± 8.8 IG = 48.1 ± 9.4	3 months

García-Rodríguez et al. [28] included 66 patients (46 women and 20 men), evenly divided between control and intervention groups, with reported average ages of 39 and 42 years, respectively. Krishnasamy et al. [29] included 88 participants (77 women and 11 men), also equally divided, with a mean age of 38.04 years. Little et al. [30] involved 818 participants, allocated to three groups: a control group (279 patients), an intervention group with dual-meetings (269 patients), and an intervention group with telematic support (270 patients), with mean ages of 52.69, 53.70, and 54.74 years, respectively. Palmeira et al. [31] included 101 women (50 control and 51 in the intervention group), with an average age of 47.8 years (47.5 control, 48.1 intervention).

Follow-up periods varied: García-Rodríguez et al. [28] and Palmeira et al. [31] followed participants for 3 months, Krishnasamy et al. [29] for 16 weeks, and Little et al. [30] for 12 months. While Palmeira et al. [31] focused on weight and BMI reduction in a Brazilian female population, Little et al. [30] examined weight variation in English patients, and García-Rodríguez et al. [28] and Krishnasamy et al. [29] investigated weight and BMI changes in mixed-gender groups. The latter [29] recruited patients from urban areas of Puducherry, while the former [28] from Tenerife.

### Quality Assessment

In analysing the methodological quality of the included studies using the Downs and Black checklist, it was observed that all four articles achieved a moderate score. Specifically, García-Rodríguez et al. scored 16, Krishnasamy et al. scored 20, Little et al. scored 23, and Palmeira et al. scored 22 (Table 2) [28–31].

**Table 2:** Qualitative analysis

	García-Rodríguez et al.	Krishnasamy et al.	Little et al.	Palmeira et al.
1. Is the hypothesis/purpose/objective of the study clearly described?	1	1	1	1
2. Are the main results to be measured clearly described in the Introduction or Methods section?	1	1	1	1
3. Are the characteristics of the patients included in the study clearly described?	1	1	1	1
4. Are the interventions of interest clearly described?	1	1	1	1
5. Are the distributions of the main confounders in each group of subjects to be compared clearly described?	0	2	1	2
6. Are the main findings of the study clearly described?	1	1	1	1
7. Does the study provide estimates of random variability in the data for the main outcomes?	1	1	1	1
8. Have any major adverse events that may be a consequence of the intervention been reported?	0	0	1	0
9. Have the characteristics of patients lost to follow-up been described?	0	0	0	0
10. Have actual probability values (e.g. 0.035 instead < 0.05) been reported for the main outcomes, except where the probability value is less than 0.001?	1	1	1	1
11. Were the subjects invited to participate in the study representative of the entire population from which they were recruited?	UTD	1	UTD	1
12. Were the subjects who were willing to participate representative of the entire population from which they had been recruited?	UTD	UTD	UTD	UTD
13. Were the staff, places and facilities where patients were treated representative of the treatment most patients receive?	1	1	1	1
14. Was an attempt made to blind the study subjects to the intervention they received?	0	0	1	0
15. Was an attempt made to blind those who measured the main outcomes of the intervention?	0	0	1	0
16. If any of the results of the study were based on “*data dredging*”, has it been clarified?	1	1	1	1
17. In clinical trials and cohort studies, do the analyses take into account the different durations of follow-up, or in case-control studies, is the time period between intervention and outcome the same for cases and controls?	1	1	1	1
18. Were the statistical tests used to assess the main findings adequate?	1	1	1	1
19. Was compliance with the intervention(s) reliable?	1	1	1	1
20. Were the main outcome measures used accurate (valid and reliable)?	1	1	1	1
21. Were patients in the different intervention groups (clinical and cohort studies) or cases and controls (case-control studies) recruited from the same population?	1	0	1	1
22. Were the study subjects in the different intervention groups (clinical and cohort studies) or the cases and controls (case-control studies) recruited in the same time period?	1	1	1	1
23. Were the study subjects randomized to intervention groups?	0	1	1	1
24. Was an attempt made to disguise the random assignment of intervention to patients and healthcare staff until the recruitment was completed and irrevocable?	0	0	1	0
25. Was there adequate correction for confounding factors in the analyses from which the main findings were drawn?	0	1	0	1
26. Have patient losses to follow-up been taken into account?	1	1	1	1
27. Did the study have sufficient power to detect a clinically significant effect where the probability value for a difference due to chance is less than 5%?	0	0	1	1
Total	16	20	23	22

UTD = unable to determine

### Telemedicine Nursing Intervention

The four studies implemented various telemedicine nursing interventions (Table 1), including phone sessions, video meetings, web-based solutions, and hybrid programs that combined different technologies. A common approach across studies was the use of web-based interventions, involving emails and websites accessible to both patients and nurses. Additionally, all studies featured phone-based interventions, such as SMS and telephone coaching, with phone calls representing the primary telemedicine method used.

García-Rodríguez et al. [28] based their intervention on phone calls and emails, supplemented by a web portal providing infographics, videos, and links to relevant resources. The control group received standard nursing care from family care units.

In the study by Krishnasamy et al. [29], the intervention group received counselling and support through both telematic and in-person modalities provided by nurses. These included phone calls, video calls, SMS, home visits, and the use of Fitbit band devices. Additionally, the intervention group participated in group educational sessions on healthy eating and physical activity, featuring lectures, group discussions, posters, and displays on healthy foods during the first two weeks. The control group received only nursing basic care, along with Fitbit monitoring.

Little et al. [30] implemented the POWeR + program, designed to teach cognitive-behavioural self-regulation strategies for weight management through physical activity and dietary regimen. Initially, patients could choose between a low-calorie diet or low-carbohydrate diet, with the flexibility to switch. Two intervention groups received web-based support: the POWeR + R group had remote nursing support, while the POWeR + F group followed a hybrid approach, combining in-person and remote meetings. The control group received minimal dietary counselling and biannual nurse follow-ups, supported by printable materials and an informational website.

Palmeira et al. [31] provided the intervention group with remote phone-based monitoring in addition to routine consultations, whereas the control group received only routine care.

The results from these studies, except the one of Little et al., are summarized in Table 3. García-Rodríguez et al. [28] reported significant reductions in obesity within the intervention group achieved at follow-up. In contrast, the control group experienced increases in anthropometric measures, though these were not statistically significant, except for the mean BMI. Krishnasamy et al. [29] observed significant reductions in obesity within the intervention group. By the end of the intervention, there were significant differences between the intervention and control groups across all anthropometric variables. In the study conducted by Little et al. [30], weight was the only parameter measured continuously throughout the study. In general, all three groups experienced weight loss over the 12-month period (Supplementary Table 1). Patients in the control group maintained an average weight loss of approximately 3 kg. Patients in the POWeR + F group lost an additional 2.54 kg (*p* < 0.001) compared to the control group, while those in the POWeR + R group lost an extra 1.97 kg (*p* = 0.002) at the 6-month follow-up (Supplementary Table 2). However, at the 12-month follow-up, no significant changes were observed compared to the previous measurement. Overall, patients in the POWeR + F group lost an average of 1.49 kg (*p* = 0.001) more than the control group, while those in the POWeR + R group had an additional weight loss of 1.27 kg (*p* = 0.007) (Supplementary Table 2).

**Table 3:** Clinical outcomes evaluated pre and post treatment

		CG			IG			
		Pre	Post	*p*-value	Pre	Post	MD (SE)	*p*-value
Garcia-Rodriguez, et al.	Weight (kg)	89.1 ± 12.4	90.2 ± 13.0	0.056	91.1 ± 13.2	88.8 ± 13.3		0.003
	WC (cm)	103.9 ± 11.9	104.5 ± 13.4	0.642	104.8 ± 11.5	103.0 ± 11.9		0.043
	BMI	31.4 ± 3.5	31.8 ± 3.6	0.046	32.4 ± 3.5	31.6 ± 3.7		0.005
Krishnasamy, et al.	Weight (kg)	79.44 ± 10.4	79.83 ± 10.44	0.72	78.72 ± 8.2	76.14 ± 8.16	2.96 (0.19)	< 0.001*
	WC (cm)	99.7 ± 8.8	99.92 ± 8.73	0.80	99.31 ± 6.3	97 ± 6.28	2.5 (0.23)	< 0.001*
	BMI	32.5 ± 4.02	32.69 ± 4.0	0.78	32.8 ± 3.35	31.69 ± 4.0	1.22 (0.07)	< 0.001*
Palmeira, et al.	Weight (kg)	93.8 ± 17.3	94.7 ± 17.7	0.041	88.8 ± 13.1	88.0 ± 13.4		0.146
	WC (cm)	106.7 ± 12.9	108.4 ± 12.7	0.107	104.2 ± 9.8	103.6 ± 10.0		0.510
	BMI	37.5 ± 6.1	37.9 ± 6.1	0.052	34.9 ± 5.2	34.7 ± 5.8		0.144

CG=control group; IG=intervention group; WC=waist circumference; BMI=body mass index; MD=mean differences between groups; SE=standard error

Data are presented as mean ± standard deviation or mean difference and standard error

* *p*-values are referred to MD= mean differences between groups post intervention

In the study conducted by Palmeira et al., a non-statistically significant decrease in BMI, weight, and WC in the intervention group was observed (Table 3). In contrast, the control group reported an increase in all variables, with only mean weight being statistically significant. However, it should also be noted that there was a loss of some participants in both groups during the follow-up.

### Meta-Analysis

The meta-analysis of weight includes 1012 patients, of whom 431 were in the intervention group. Initially, 1352 patients were enrolled in the pre-evaluation, but only those who completed the intervention were considered in the analysis. The total number of patients included in the meta-analyses of BMI and WC was 255, with 128 patients in the intervention group and 127 in the control group.

### Weight

**Fig. 2:**
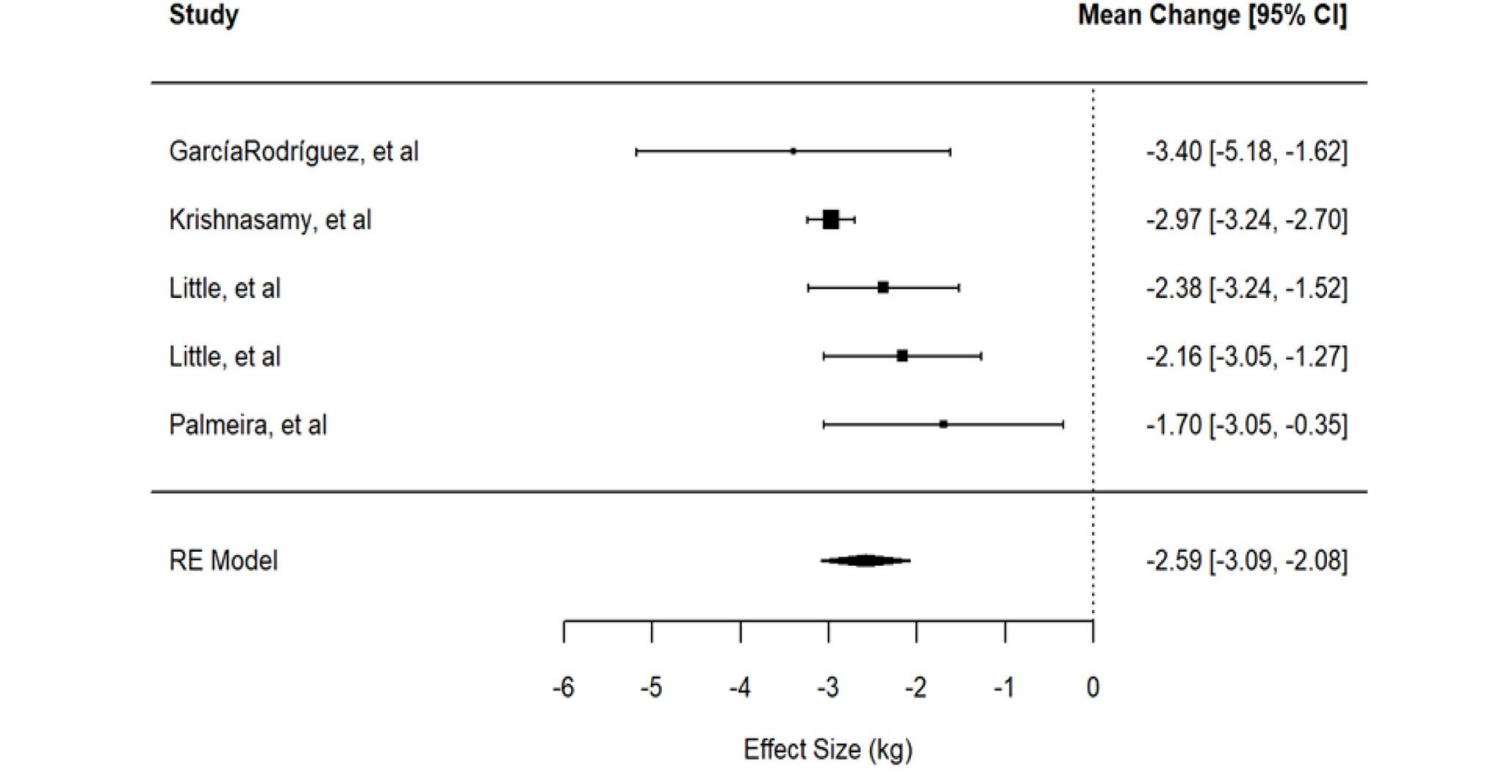
Forest plot for the effect of the protocols on weight reduction

For measuring the impact of the protocols on weight loss, we first considered a model where all arms of the intervention of Little et al. [30] were included, as they were two independent, different treatments. The estimated common effect, using the classical two-level model, is -2.59 kg, supporting the effectiveness of the intervention in promoting weight loss. The 95% CI associated with the point estimate is [-3.09, -2.08]. The hypothesis of no difference between the treatment and the control groups is rejected both using standard methods (*p* < 0.001) and when considering correction methods for small study number (*p* = 0.001). The estimated variability due to between-study heterogeneity is 44% of the total variability (95% CI between 0 and 94.87), while the estimate of the variance between studies is 0.14, corresponding to standard deviation between the studies equal to 𝜏=0.368 kg with 95% confidence interval in (0, 1.178). The Cochrane statistics test for heterogeneity results equal to Q (df = 3) = 4.987, *p* = 0.173. These results suggest that there is no evidence of marked heterogeneity. The large confidence intervals for the heterogeneity estimates are due to the small number of studies. The forest plot is reported in Fig. 2.

For this outcome, we also performed some sensitivity analysis, including in the pool of studies only one arm of the study of Little et al. [30] at a time. This is because the effect measure depends on a common control group, thus the assumption of independence of the two effect sizes is not satisfied. Under the two models with 4 studies, the effects are − 2.61 kg with 95% CI [-3.24, -1.97] and − 2.70 kg, with 95% CI [-3.24, -2.15], in agreement with the findings.

### BMI

**Fig. 3:**
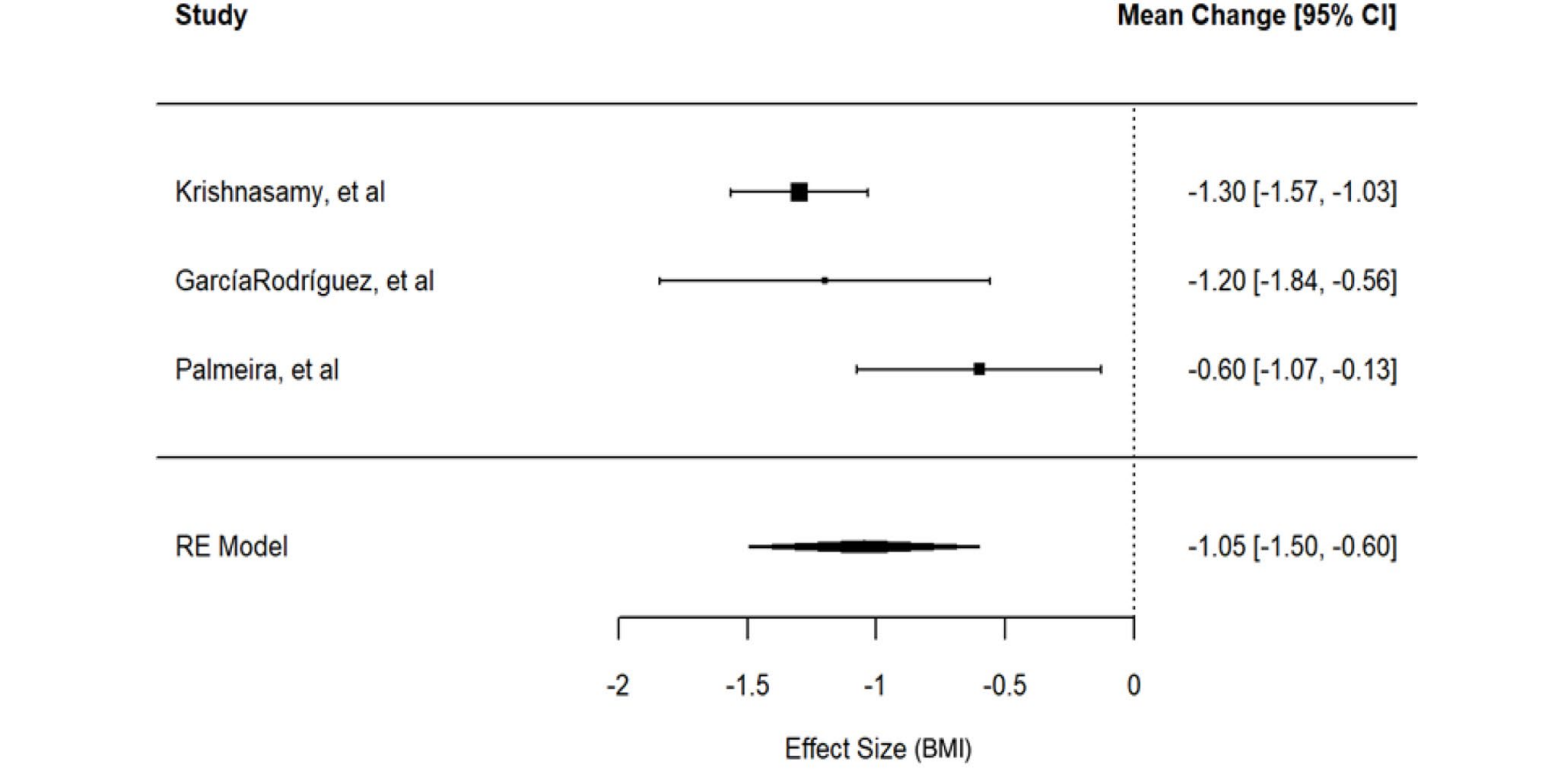
Forest plot for the effect of the protocols on BMI reduction

We fitted a two-level model considering the three available studies [28, 29, 31]. The estimated loss in BMI score obtained by the meta-analytic model was 1.05 kg/m^2^ (95% CI [-1.50, -0.60]). This result confirms that overall, the telenursing intervention reduced the BMI over that of patients in the treatment group. The *p*-value for the hypothesis of no difference of the two arms is < 0.001, which leads us to reject this hypothesis at the usual level. Using the Skovgaard method, for controlling the small number of studies, a *p*-value equal to 0.0338 was obtained, confirming the evidence of improvement. The $\:{I}^{2\:}=\:67.07\:\%$ with 95% CI $\:\left[0.00\%,\:99.10\%\right]$ indicates substantial heterogeneity between the studies’ estimates. The estimated value of $\:{\tau\:}^{2\:}=0.1$, corresponding in the standard deviation scale to 0.32 points of BMI with 95% CI in [0, 2.36], suggests that the between study heterogeneity is in absolute terms negligible. The uncertainty around this estimate is due to the small number of studies. Although the decision to use a random-effects model was made a priori, the Cochran statistic supports this choice, Q (df = 2) = 6.392, *p* = 0.041. It is also worth noting that while the confidence intervals for the $\:{I}^{2}$and $\:{\tau\:}^{2\:}$ statistics include values close to zero, these numbers are small but not exactly zero. The forest plot is reported in Fig. 3.

### Waist Circumference

**Fig. 4:**
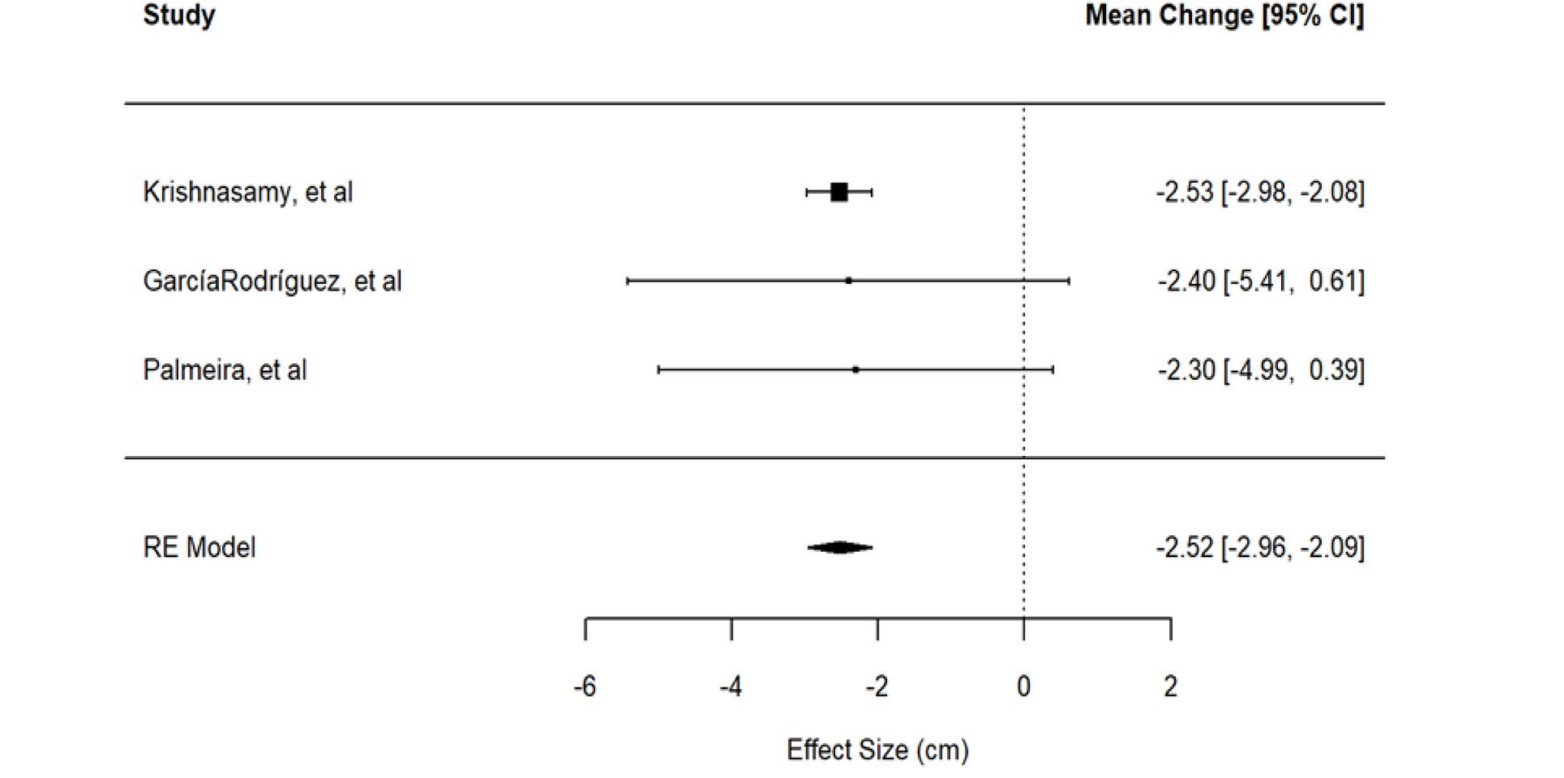
Forest plot for the effect of the protocols on waist circumference

A two-level meta-analytic model was considered using data on the effect of the protocol on WC [28, 29, 31]. The model revealed an absence of heterogeneity among the studies, ($\:{\tau\:}^{2}$=0 and $\:{I}^{2}$=0, confidence intervals equal to the null set; Cochrane statistics Q (df = 2) = 0.034 with *p* = 0.983). Thus, a fixed effect model was fitted to the data. The meta-analysis yielded an estimated reduction in WC of -2.52 cm (95% CI: [-2.96, -2.09]). The hypothesis of no difference in WC between the two groups was rejected at the conventional significance level (*p* < 0.001). The *p*-value obtained with the Skovgaard method, which adjusts for the small number of studies, was 0.0121, confirming the significance of the intervention. Fig. 4 presents a forest plot for visualizing these results.

## Discussion

A total of 4 studies with moderate quality were included and each study highlighted the significant benefit of nursing telemedicine in weight loss and obesity management, particularly when these interventions were implemented consistently over extended periods [32]. Importantly, the meta-analysis confirmed the effectiveness of these interventions in reducing BMI, WC, and body weight. The pooled estimate from three studies [28, 29, 31] demonstrated a significant reduction in BMI. The Skovgaard method validated the robustness of these findings. Similarly, WC reduction showed an estimated decrease of -2.52 cm with no heterogeneity. The weight reduction analysis, which included data from four studies, demonstrated an overall weight loss of -2.59 kg, supporting the efficacy of these interventions. Sensitivity analyses further reinforced these findings, ensuring that the results were not driven by the failure to make an independence assumption between effect sizes from Little et al. [30].

The studies analysed employed a wide range of telemedicine approaches, from simple telephone support to video conferencing and the use of wearable devices for remote patient monitoring. This variety reflects telemedicine’s adaptability, allowing for the customization of interventions based on patients’ specific needs and available resources [33]. However, it was clearly shown that the effectiveness of these interventions increases when digital tools are combined with traditional methods, such as face-to-face meetings with nurses, underscoring the importance of maintaining direct human contact even in telemedicine-based care [32].

One of the main advantages of telemedicine is its ability to provide continuous monitoring and patients education in a flexible and consistent manner [34]. This feature is evident in the study by García-Rodríguez et al. [28], where the telemedicine platform improved patient adherence to lifestyle interventions, leading to significant reductions in body weight and BMI. Similarly, Krishnasamy et al. [29] demonstrated the efficacy of telemedicine in improving parameters such as abdominal circumference and blood pressure, as well as fostering healthier eating habits in the intervention group.

However, the sustainability of these interventions over the long-term remains a key challenge. Although data from Little et al. [30] showed that telemedicine interventions can maintain a 5% weight loss from baseline even at 12 months, the difficulty of maintaining adherence to remote programs remains a concern. Continuous patient engagement appears to be one of the greatest challenges of telemedicine, as demonstrated in Krishnasamy et al.‘s [29] study, where patients initially showed enthusiasm but struggled to maintain commitment to physical activity and healthy eating. Similarly, Palmeira et al. [31] reported a significant dropout rate, with nearly 20% of patients leaving the study over three months (from 101 to 81 participants). These findings reflect broader concerns noted in other reviews on the long-term efficacy of telemedicine for obesity management, pointing out the need for strategies that enhance sustained engagement [15].

An important aspect that emerged is the role of structured health education in telemedicine interventions. Interventions grounded in well-defined theoretical approaches - such as the cognitive-behavioural approach used by Little et al. [30], or motivational models adopted by García-Rodríguez et al. [28], - can help patients to develop self-regulation skills and adopt more effective and lasting dietary and behavioural changes. Notably, interventions that combine health education with strong motivational support, such as those based on motivational coaching techniques and active patient involvement, appear to be more successful. Additionally, the implementation of dietary regimen choices by participants in the study by Little, et al. [30] demonstrated greater effectiveness in terms of weight reduction.

Another key consideration is the accessibility and flexibility of telemedicine, which can overcome geographical and logistical barriers that traditionally limit access to healthcare services, particularly for patients with mobility difficulties or those living in rural areas. Nevertheless, as noted by Krishnasamy et al. [29], access to technology and family involvement can affect the effectiveness of interventions. Therefore, to maximize the benefits of telemedicine, it is essential to ensure technological accessibility and tailor interventions to the socioeconomic and cultural needs of patients. In this context, nurses play a key role in supporting patients in acquiring the necessary digital skills and resources, thus promoting equitable access to healthcare services [29].

The implementation of telemedicine in the management of overweight and obesity has significant implications for the nursing profession, reshaping care delivery methods, required skills/competencies, and the nurse-patient relationship. Nurses assume a more central role in remote health management, monitoring patients through technological devices, digital platforms, and remote monitoring systems. This strengthens interactions between nurses and patients beyond traditional in-person visits, expanding nurses’ professional responsibilities through tools such as video calls, emails, and telemonitoring platforms [35]. Through telemedicine, nurses can monitor real-time parameters such as weight, BMI, physical activity levels, and patients’ dietary habits. Additionally, nurses are expected to intervene promptly in case of clinical concerns or emergencies, and to coordinate interventions with other healthcare professionals to ensure a comprehensive and timely care [36]. As health education shifts to the virtual setting, nurses are expected to adapt educational strategies to effectively support behavioural change, providing consistent and personalized guidance on diet, exercise, and lifestyle changes. These activities require the development of advanced skills in education and counselling, including patient counselling and motivational support, to foster autonomy and treatment adherence [37].

To work effectively with telemedicine, nurses should develop advanced technological skills. They need to become proficient in using health apps, secure communication platforms, and telemonitoring tools, while also managing data security and patient privacy. This strengthens their digital readiness, ensures regulatory compliance, and protects patient information [38]. Telemedicine also fosters the creation of multidisciplinary teams, encouraging collaboration between nurses, doctors, dietitians, psychologists, and other specialists. Data sharing can facilitate a more coordinated and integrated approach to obesity management among professionals, enhancing the effectiveness of interdisciplinary interventions [35]. Furthermore, nurses’ constant presence in telemedicine can provide ongoing emotional support to patients, reducing stress associated with obesity management and reinforcing motivation through regular contact [39]. Finally, nursing telemedicine can be valuable not only in managing obesity but also in addressing other conditions or aspects of care. As supported by the literature - particularly in the aftermath of the COVID-19 pandemic, when nurse leadership has had to re-establish consistent communication with staff to ensure effective teamwork, high-quality patient care, and smooth operations [40], telemedicine has demonstrated its potential, although its implementation in current practice remains limited [41].

This study has several limitations that should be considered when interpreting the findings. Only four moderately rated studies met the inclusion criteria, limiting the overall strength and generalizability of the conclusions. Despite this, a second-order asymptotic approach—known for improving accuracy in analyses involving small samples—was employed to strengthen the reliability of the meta-analytic results. A second limitation is the considerable variability in the content and delivery of the telemedicine interventions, which makes it challenging to draw definitive conclusions about the most effective nursing approaches. Additionally, the short follow-up durations at the patient level limit the ability to assess long-term outcomes. These findings, while promising, underscore the need for further high-quality research to establish standardized, evidence-based telemedicine protocols in nursing care for obesity management.

## Conclusion

This systematic review with meta-analysis suggests that nursing telemedicine interventions can effectively support short-term weight reduction and adherence to lifestyle changes.

By leveraging digital tools—such as web platforms, mobile apps, and remote monitoring—these interventions offer continuous, personalized support that enhances patient adherence to dietary and physical activity goals. Nursing telemedicine interventions can reach underserved or remote populations who may have limited access to traditional in-person care, promoting equity in healthcare delivery. This is particularly important in policymaking, as socioeconomic factors play a critical role in obesity rates, with higher prevalence observed among individuals with lower educational level and economic status. Furthermore, given the link between obesity and chronic conditions like diabetes and hypertension, integrating effective telemedicine interventions into public health strategies could significantly reduce long-term healthcare costs and improve population health outcomes.

Despite these benefits, challenges remain, including disparities in technology access, varying levels of patients’ digital literacy, and the need for specialized nurse training. Additionally, most of the studies reviewed have focused on short-term effects, highlighting the need of high-quality research with larger sample sizes and extended follow-up. Future research should refine intervention protocols, optimize interaction frequency, and explore factors influencing treatment success. Additionally, prioritizing personalized care may enhance patient responsiveness and long-term adherence. Finally, investing in nurse training and developing telemedicine-specific competencies will be crucial to ensuring the quality and sustainability of these interventions for obesity and overweight management.

## Supplementary Information

Below is the link to the electronic supplementary material.

Supplementary Material 1

## Data Availability

No datasets were generated or analysed during the current study.

## Key References

* Importance reference, ** Very important reference.

**Kupila SKE, Joki A, Suojanen LU, Pietiläinen KH. The Effectiveness of eHealth Interventions for Weight Loss and Weight Loss Maintenance in Adults with Overweight or Obesity: A Systematic Review of Systematic Reviews. *Curr Obes Rep.* 2023 Sep;12(3):371–394. 10.1007/s13679-023-00515-2. Erratum in: *Curr Obes Rep.* 2023 Dec;12(4):544–545. 10.1007/s13679-023-00530-3.

This review of systematic reviews highlights that eHealth interventions are effective for weight loss in adults with overweight or obesity, especially when combined with professional support. Key features include personalization, feedback, and self-monitoring. However, evidence on long-term weight loss maintenance remains limited.

**Sen A, Ho KYA, Goh WKF, Chew HSJ. The roles of nurses in preventing and managing excess weight among adults: A systematic scoping review. *Nurs Outlook.* 2025 Mar-Apr;73(2):102377. 10.1016/j.outlook.2025.102377.

A scoping review discussing the role of nursing in managing excess weight in adults.

*Piwowarczyk E, MacPhee M, Howe J. Nurses’ Role in Obesity Management in Adults in Primary Healthcare Settings Worldwide: A Scoping Review. *Healthcare (Basel)*. 2024 Aug 26;12(17):1700. 10.3390/healthcare12171700.

Nurses play a key role in primary care obesity management, focusing on lifestyle interventions, assessments, education, care coordination, and patient support.

*Clement David-Olawade A, Olawade DB, Ojo IO, Famujimi ME, Olawumi TT, Esan DT. Nursing in the Digital Age: Harnessing telemedicine for enhanced patient care. *Inform Health*. 2024;1:100–10. 10.1016/j.infoh.2024.07.003.

Telemedicine is associated with better patient outcomes and increased satisfaction. Nurses play a key role in teletriage and remote monitoring, while teleconsultations improve care efficiency and access.
