# Supplementary material for: Effectiveness of Telemedicine Nursing Interventions in the Management of Overweight and Obesity in Adults: A Systematic Review and Meta-Analysis
Source: Curr Obes Rep. 2025 Jul 30;14(1):63. doi: 10.1007/s13679-025-00655-7 (PMC12310891; doi:10.1007/s13679-025-00655-7)
Supplement: Supplementary file 1 — Supplementary Material 1 [file 13679_2025_655_MOESM1_ESM.docx]

**Supplementary Table 1. Weight and BMI at baseline, 6 months, and 12 months after the intervention.**

| **Little, et al** |  | **CG** | | | **POWeR+FF** | | | **POWeR+R** | | |
| --- | --- | --- | --- | --- | --- | --- | --- | --- | --- | --- |
|  |  | **Pre** | **Post**  **6 months** | **Post**  **12 months** | **Pre** | **Post**  **6 months** | **Post**  **12 months** | **Pre** | **Post**  **6 months** | **Post**  **12 months** |
|  | **Weight (kg)** | 104.38±21.11 | 101.91±19.35 | 101.73±19.57 | 102.40±16.87 | 97.55±15.99 | 98.56±15.95 | 102.93±18.26 | 98.30±18.34 | 99.72±18.88 |
|  | **BMI** | 37.10 ± 5.97 |  |  | 36.66 ± 5.36 |  |  | 36.28 ± 5.65 |  |  |

CG=control group; BMI = body mass index; POWeR+F=POWeR+ group with dual meetings; POWeR+R=POWeR+ group with remote meetings

Data are presented as mean ± standard deviation

**Supplementary Table 2. Difference in weight loss (kg) for POWeR+ vs CG, with imputed data (50 replications).**

| **Little, et al** | **POWeR+FF** | | | **POWeR+R** | | |
| --- | --- | --- | --- | --- | --- | --- |
|  | **Post**  **6 months** | **Post**  **12 months** | **Overall** | **Post**  **6 months** | **Post**  **12 months** | **Overall** |
|  | - 2.54 (-3.66 to -1.42)  p<0.001 | -0.37 (–1.66 to 0.92)  p=0.566 | -1.49 (–2.41 to –0.58)  p=0.001 | -1.97 (–3.18 to –0.76)  p=0.002 | -0.58 (–1.88 to 0.72)  p=0.375 | -1.27 (–2.19 to –0.34)  p=0.007 |

CG=control group; POWeR+F=POWeR+ group with dual meetings; POWeR+R=POWeR+ group with remote meetings

Data are presented as difference in weight loss (95% CI)
